# Le méningiome du sinus maxillaire: à propos d'un cas avec revue de la littérature

**DOI:** 10.11604/pamj.2014.18.137.4746

**Published:** 2014-06-12

**Authors:** Imane Mezouri, Sara Bellefqih, Hanane Chenna, Hanan El Kacemi, Tayeb Kebdani, Noureddine Benjaafar

**Affiliations:** 1 Service de Radiothérapie, Institut National d'Oncologie, avenue Allal Elfassi, Rabat, Maroc

**Keywords:** Méningiome, extra crânien, sinus maxillaire, radiothérapie, cancer, Meningioma, extra cranial, maxillary sinus, radiotherapy, cancer

## Abstract

Les méningiomes extracrâniens sont rares, leur localisation au niveau du sinus maxillaire est exceptionnelle. Nous rapportons le cas d'une patiente âgée de 41 ans, ayant présenté une exophtalmie avec des céphalées intermittentes évoluant depuis 8 ans. Le diagnostic a été retenu à partir de la biopsie et du scanner cérébral et du massif facial. Le traitement a consisté en une irradiation exclusive à la dose de 54 Gray (Gy). La patiente est restée en bon contrôle locorégional, après un recul de 18 mois. A travers ce cas clinique on a démontré que le sinus maxillaire peut être touché par le méningiome, et qu'il doit être inclus dans le diagnostic différentiel des tumeurs des tissus mous.

## Introduction

Les méningiomes sont des tumeurs bénignes extra parenchymateuses développées à partir des cellules méningées de l'arachnoïde, ils représentent 13 à 26% des tumeurs primitives cérébrales. Les méningiomes extracrâniens sont rares et ils touchent principalement l'orbite, l'os temporal, les fosses nasales et la mandibule. Leur localisation au niveau du sinus maxillaire est exceptionnelle. Nous rapportons un rare cas du méningiome du sinus maxillaire gauche, récusé chirurgicalement et traité par radiothérapie exclusive

## Patient et observation

**Figure 1:**
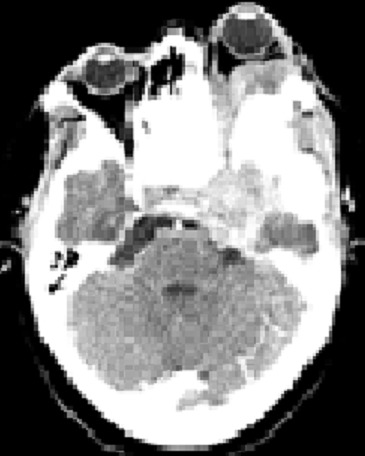
Coupe scannographique axiale montrant un processus tissulaire au dépends du sinus maxillaire gauche localement avancé, responsable d'une exophtalmie en regard

Il s'agit d'une patiente âgée de 41 ans, sans antécédents pathologiques particuliers qui a consulté en consultation neurochirurgie pour une exophtalmie avec des céphalées intermittentes évoluant depuis 8 ans. Une tomodensitométrie (TDM) cérébrale et du massif facial a objectivé un processus tissulaire agressif au dépends du sinus maxillaire gauche s’étendant au cavum, fosses nasales gauches, la base du crâne ainsi que l'orbite gauche avec exophtalmie au niveau du parenchyme cérébral en regard (Figure 1).

**Figure 2:**
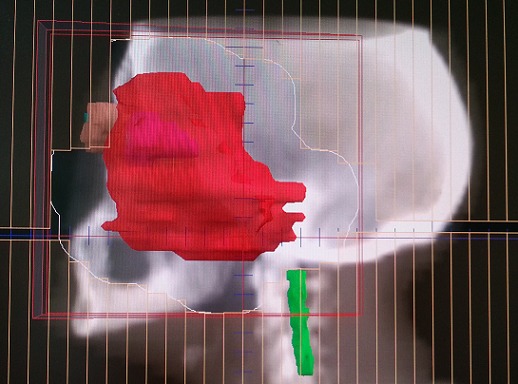
Image issue du système de planification du traitement pour radiothérapie montrant un champ latéral gauche. Le volume cible correspond au contour rouge

**Figure 3:**
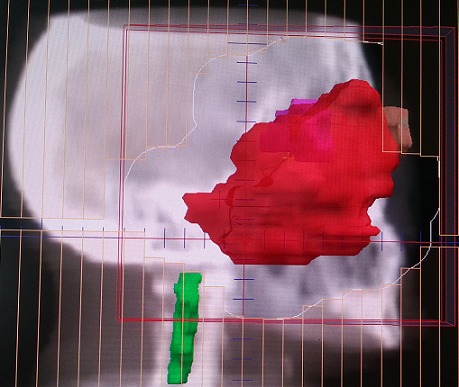
Image issue du système de planification du traitement pour radiothérapie montrant un champ latéral droit

**Figure 4:**
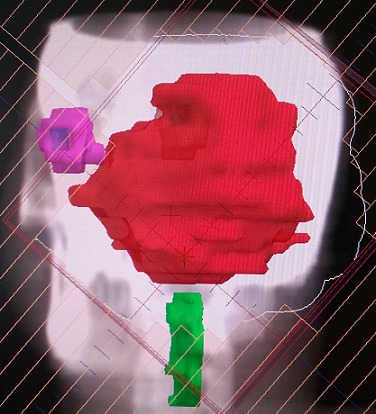
Image issue du système de planification du traitement pour radiothérapie montrant un champ antérieur

Une biopsie du cavum a été réalisée, l’étude histologique a montré l'aspect du méningiome méningothélial. En réunion de concertation pluridisciplinaire, la patiente a été récusée chirurgicalement et il a été décidé de réaliser une radiothérapie exclusive. Ainsi, on a réalisé un scanner dosimétrique. Les images scannographiques ont été transférées vers la console de contourage, la tumeur macroscopique a été contournée. Une balistique par trois champs (deux latéraux opposés et un antérieur) a été planifiée (Figure 2, Figure 3, Figure 4). La dose totale prescrite était de 54 Gray (Gy) avec un fractionnement de 2Gy/fraction, une fraction par jour, 5jour/7jours; Le traitement s'est étalé sur 38 jours. Après un recul de 18 mois, la patiente est restée en bon contrôle locorégional.

## Discussion

Les méningiomes sont décrits chez les adultes, avec une tranche d’âge entre 20 et 60 ans, avec un pic à l’âge de 40 ans. Une prédominance féminine, particulièrement dans les méningiomes de la moelle épinière, est très élevée [1, 2] Bien que les méningiomes puissent potentiellement toucher n'importe quel site dans les méninges, certains emplacements intracrâniens sont les plus fréquents: au niveau parasagittale et la faux du cerveau. D'autres peuvent siéger au niveau des convexités cérébrales, le carrefour olfactif, la selle turcique, l'angle ponto-cérébelleux et le clivus. La combinaison intra et extra crânien est décrite dans 20% des cas [1, 2]

Le méningiome extracrânien est une tumeur rare, il représente seulement 2% des méningiomes. Quatre formes des méningiomes extracrâniens ont été suggérées [3]: 1. Méningiome intracrânien primitif avec extension extracrânienne; 2. Extension extracrânienne d'un méningiome siégeant dans le foramen nerveux; 3. Méningiome ectopique sans aucun rapport avec un foramen d'un nerf crânien ou des structures intracrâniennes; 4. Métastase d'un méningiome intracrânien. Les sites extra crâniens les plus rapportés dans la littérature sont la cavité nasale, les sinus para nasaux, le crâne, le scalp, les tissus mous de la face et du cou et la glande parotide [4–6].

L’étiopathogénie des méningiomes extra crâniens réside dans la migration des cellules arachnoïdiennes de la crête neurale. Différents mécanismes ont été suggérés comme la migration des cellules arachnoïdiennes des gaines nerveuses à partir du foramen crânien; déplacement des corps de Pacchioni en extracrânien pendant le développement embryologique; ou déplacement des îlots arachnoïdiens des cellules mésenchymateuses non différenciées à cause d'un traumatisme ou d'une hypertension intracrânienne [7].

Les symptômes sont liés à la taille tumorale, sa localisation et son profil de croissance. Sur le plan histologique, ces lésions sont indiscernables des méningiomes intracrâniens. Plusieurs classifications ont été établies; elles se basent sur la morphologie cellulaire dominante. Le méningiome peut se présenter comme méningothélial, transitionnel, fibroblastique, angiomateux, atypique et anaplasique [8]. Dans notre cas, le diagnostic histologique était un méningiome méningothélial. Il n y'a aucune technique radiologique spécifique pour ces tumeurs [9]. Dans notre cas, la patiente a bénéficié d'une TDM cérébrale.

La résection chirurgicale complète est le traitement principal des méningiomes, cependant leur localisation peut la rendre difficile voir impossible. La radiothérapie jour alors un rôle important comme traitement complémentaire en cas de résection incomplète ou exclusive dans le cas des tumeurs non ré-sécables [9, 10]. Notre patiente a bénéficié d'une radiothérapie exclusive à une dose de 54 Gy.

## Conclusion

En conclusion, Le méningiome du sinus maxillaire est une tumeur inhabituelle, il devrait être inclus dans le diagnostic différentiel des tumeurs de tissu mous. Il doit être suspecté devant la présentation d'une tuméfaction, à évolution insidieuse, siégeant au niveau de la région maxillaire. La chirurgie reste son traitement principal.
